# LncRNA在非小细胞肺癌中的研究进展

**DOI:** 10.3779/j.issn.1009-3419.2016.02.10

**Published:** 2016-02-20

**Authors:** 黎明 樊, 志东 胡

**Affiliations:** 300052 天津，天津医科大学总医院医学检验科 Department of Medical Clinical Laboratory, Tianjin Medical University General Hospital, 300052 Tianjin, China

**Keywords:** 长链非编码RNA, 肺肿瘤, 基因调控, LncRNA, Lung neoplasms, Gene regulation

## Abstract

肺癌相关死亡是世界范围内癌症导致死亡的最常见原因。非编码RNA无蛋白编码功能，但可发挥多种生物学作用。长链非编码RNA是最新表征的一种至少包含200个核苷酸且不编码蛋白的RNA。多种长链非编码RNA具有促进或抑制肿瘤进展的功能，包括非小细胞肺癌。由于他们在调节基因表达方面的基本作用并涉及肿瘤发生的生物学机制，有望成为以组织或血液为基础的癌症生物标志物。在该综述中，我们着重强调lncRNA在非小细胞肺癌中的作用，并讨论其作为诊断及预后标志物、治疗靶标的潜在临床应用。

## Abstract (en)

Lung cancer related death is the most common cause of cancer death worldwide. Non-coding RNA has no protein coding capacity, but can play roles in a variety of biology. Long non-coding RNA (lncRNA) is at least 200 nt long. A variety of lncRNAs could promote or inhibit tumor development and progression in many tumors, including non-small cell lung cancer (NSCLC). Due to their fundamental role in regulating gene expression, they involve in biological mechanism of the tumor, and so they are expected to be new cancer biomarkers. In this review, we emphasized the roles of lncRNAs in NSCLC, and discuss them as diagnostic and prognostic markers and as therapeutic targets.

肺癌是常见的恶性肿瘤，肺癌相关死亡是世界范围内癌症导致死亡的最常见原因。根据不同的分化程度和形态特征，肺癌分为小细胞肺癌（small cell lung cancer, SCLC）及非小细胞肺癌（non-small cell lung cancer, NSCLC），其中NSCLC占所有肺癌患者的85%左右^[1]^。

人类基因组测序计划显示仅有2%的基因编码蛋白，有超过90%的基因转录为RNA，并未编码为蛋白质^[2]^。这些非编码RNA仅有非常局限或无蛋白编码功能，可分为两种类型：管家ncRNA（housekeeping non-coding RNA）和调节ncRNA（regulatory non-coding RNA）。调节ncRNA以空间或时间特异性模式进行表达。根据大小，调节ncRNA可被进一步分为两个亚型：转录子少于200 nt的非编码性小RNA及转录子介于200 nt至100 kb间的长链非编码RNA（long non-coding RNA, lncRNA）。非编码性小RNA包括微小RNA（microRNA, miRNA）、核仁小RNA（small nucleolar RNA, snoRNA）、小分子干扰RNA（small interfering RNA, siRNA）、小核RNA（small nuclear RNA, snRNA）等。LncRNA之前一直被认为是基因组转录过程中的“噪音”，不具有生物学功能。随着近年来研究的深入，人们发现lncRNA参与了表观遗传、转录及转录后调控等多种途径的基因调控，成为近些年研究的热点。本文主要结合lncRNA的概念及功能机制，探讨lncRNA在NSCLC中的最新研究进展。

## 1 LncRNA的特点及生物学特性

LncRNA为至少包含200个核苷酸的功能性RNA，由Okazaki等^[3]^首次在小鼠的DNA转录产物中发现。已有的研究^[4]^表明，lncRNA的产生主要有以下几种途径：①蛋白编码基因的结构中断，从而形成一段lncRNA；②一个独立基因与两个未转录基因串联，产生含多个外显子的lncRNA；③局部的复制子串联形成；④非编码RNA在复制的过程中反移位产生；⑤基因中插入一个转座成分而产生有功能的非编码RNA。根据lncRNA的位置和来源，可将其分为内含子lncRNA（intronic lncRNA）、基因间lncRNA（intergenic lncRNA）、正义lncRNA（sense lncRNA）、反义lncRNA（antisense lncRNA）和双向lncRNA（bidirectional lncRNA）。

LncRNA通常较长，具有mRNA样结构，很多lncRNA由RNA聚合酶Ⅱ转录而来，有些具有poly（A）尾巴和启动子结构，分化过程中有动态的表达与不同的剪接方式。在植物、动物、原核生物及病毒中均可发现lncRNA具有组织特异性：不同组织之间的lncRNA表达量不同。同时，lncRNA具有时空特异性：同一组织或器官的不同生长阶段，lncRNA表达量也会变化。

## 2 LncRNA在NSCLC中的作用

LncRNA在肿瘤发生中发挥多种作用，因此，对于阐释癌症的进展来说，癌症相关lncRNA的鉴定及与之相关的生物学功能和分子机制的探究非常重要。越来越来的证据显示，lncRNA参与NSCLC的进展。肿瘤抑制lncRNA的研究为阐释NSCLC的发病和发展提供了一条新的路径，同时为寻求更多有效治疗NSCLC的药物提供了新的平台。这里，我们将介绍几个与NSCLC有关的lncRNA并讨论其在诊断、预后及治疗中的潜在临床应用。

### 2.1 癌症相关lncRNA

多个生物过程参与肿瘤进展的精确调节，这些过程涉及激活癌基因或沉默肿瘤抑制基因。NSCLC的启动也不例外。癌基因为产物可促进肿瘤启动和进展的基因。癌基因的激活在人类肿瘤的发病中发挥重要作用。因此，发现新的癌基因并研究其功能对于开发新的治疗药物非常重要。LncRNAs可分为致癌lncRNA和肿瘤抑制lncRNA。肺腺癌转移相关转录子1（metastasis-associated lung adenocarcinoma transcript 1, MALAT1）、结肠癌相关转录子2（colon cancer-associated transcript 2, CCAT2）、HOTAIR及AK126698为致癌lncRNAs，他们的过表达可促进细胞生长、迁移和侵袭，促进NSCLC的进展。

由于诊断技术有限，且癌症意识薄弱，NSCLC诊断时通常已为晚期，这导致其5年生存率不理想。为了改善生存率，亟需发现有效的诊断和预后生物标志物。鉴于lncRNA在肿瘤进展中发挥的多种作用^[5]^，lncRNA作为生物标志物的相关研究亦日趋增多。目前，仅发现数个lncRNA在患者的体液中可作为候选的生物标志物^[6]^，如HULC在肝细胞癌中呈高表达^[7]^，在前列腺癌患者的粘液中可发现前列腺癌基因3（prostate cancer gene 3, PCA3），且准确性高^[8]^。MALAT1可作为NSCLC诊断的生物标志物，MALAT1预示NSCLC的不良预后且可诱导迁移及肿瘤生长^[9]^。

#### 2.1.1 MALAT1

MALAT1，又称核富集常染色体转录产物2（nuclear-enriched transcript 2, NEAT2），是高度保守的lncRNA，转录子大小为8.7 kb，位于染色体11q13。Tripathi等^[10]^认为在不同的细胞类型和细胞周期中MALAT1的异常表达发挥“分子海绵”的作用。

MALAT1在多种肿瘤中呈过表达，包括乳腺癌、前列腺癌、直肠癌、肝癌及NSCLC，特别在早期转移的患者中。Ji等^[9]^研究发现MALAT1在早期NSCLC中可预测转移，其表达与NSCLC患者的转移密切相关。与Ji等^[9]^的研究一致，Schmidt等^[11]^发现MALAT1可刺激迁移、侵袭及肿瘤生长，但其潜在的机制尚未被阐释。其原因可能为在特定的细胞中MALAT1的异常表达可导致异常的选择性剪接，导致基因的错误表达，如致癌转录因子B-MYB^[12]^。同时，他们的研究^[11]^认为MALAT1参与NSCLC的发展，故MALAT1的过表达可作为识别肿瘤是否具有转移潜质的标志，同时其也是第一个被识别的可调节NSCLC转移的lncRNA。

已有的研究发现，MALAT1可作为NSCLC的诊断性生物标志物。Weber等^[13]^检测了45例NSCLC患者及25例健康人外周血中MALAT1的表达水平，他们发现在NSCLC患者中很容易检测到MALAT1，且MALAT1满足作为诊断生物标志物的多个重要特征：容易获取、微创手段即可获得样本且具有高特异性。因此，他们建议MALAT1用作NSCLC的诊断生物标志物。但同时也具有两个缺陷：①MALAT1的敏感性并不十分令人满意，因此，他不能单独被用作标志物，但可作为互补的标志物。②癌症患者与健康人中MALAT1的表达水平差异显著，但腺癌（adenocarcinoma, AdCa）与鳞癌（squamous cell carcinoma, SCC）间差异不明显，这提示在区别AdCa与SCC方面MALAT1不具诊断价值。

除作为诊断生物标志物外，MALAT1还预示NSCLC患者的预后不良。Schmidt等^[11]^研究发现MALAT1联合胸腺素β4（thymosin β4）可作为早期NSCLC患者生存的独立预后因素，MALAT1的过表达与NSCLC患者的不良预后有关。

#### 2.1.2 CCAT2

研究^[14, 15]^发现，CCAT2在结直肠癌和乳腺癌中呈过表达。最近，Qiu等^[16]^研究发现CCAT2在NSCLC中表达上调，通过siRNA沉默CCAT2可抑制体外NSCLC细胞系的增殖和侵袭。至于潜在的机制，Qiu等^[16]^未作解释。然而，Ling等^[14]^发现CCAT2可通过TCF7L2介导的转录上调MYC、miR-17-5p及miR-20a的表达，且CCAT2过表达细胞中miR-17-5p或miR-20a的敲除可导致细胞迁移能力的大幅下降。此外，CCAT2与TCF7L2的相互作用可增加Wnt的信号活动。因此我们认为CCAT2通过相同的通路在NSCLC中发挥作用。具体机制的阐释仍需大量研究。

CCAT2在NSCLC中呈高表达，其具有作为诊断生物标志物的潜力。CCAT2与MALAT1不同，CCAT2在肺腺癌（lung adenocarcinoma, LAD）中呈特异性过表达，而MALAT1在LAD及SCC均高表达。因此，CCAT2可作为区分LAD与SCC的生物标志物。同时，Qiu等^[16]^研究发现CCAT2联合血清癌胚抗原（carcino-embryonic antigen，CEA）可显著提高预测效率，该联合可预测NSCLC中的淋巴结转移。然而，Qiu等^[16]^研究同时发现，由于淋巴结阴性NSCLC中CCAT2的表达水平并不明显，故CCAT2不可单独作为淋巴结转移的生物标志物。

#### 2.1.3 HOTAIR及AK126698

对于NSCLC患者来说，手术通常为治疗的第一选择。然而由于诊断时已处于晚期，多数患者已失去最佳的手术时机。因此，化疗在NSCLC的治疗中发挥重要作用。目前，以铂类为基础的化疗是NSCLC的一线化疗方案。在所有的铂类药物中，顺铂应用最为广泛。然而，多数患者对顺铂并不敏感，这严重阻碍了化疗的效率。研究化疗耐药机制对于获得较好的化疗疗效非常重要。

HOTAIR为长度为2.2 kb的lncRNA^[17]^，位于染色体12q13.13。他是第一个被发现的涉及肿瘤发生的lncRNA，可通过募集多硫抑制性复合体2（polycomb repress complex 2, PRC2）或重组染色质促进各种肿瘤的进展^[18]^。另外，HOTAIR也参与NSCLC中的顺铂的化疗耐药。近期，有研究^[19]^显示HOTAIR的过表达与LAD细胞对顺铂的敏感性有关。在体外，研究发现与亲代A549细胞相比，顺铂耐药的A549/DDP细胞中HOTAIR呈高表达，敲除HOTAIR可恢复A549/DDP细胞对顺铂的敏感性。在体内，在顺铂敏感的LAD组织中HOTAIR的表达明显下调。研究发现HOTAIR与PRC2及LSD1/CoREST/REST复合体相互作用，将其招募至HOXD基因簇，使部分基因沉默。表观遗传沉默是肿瘤抑制基因失活的常规机制。Zeste 2增强子（enhancer of Zeste 2, EZH2）是PRC2的组分，介导通过组蛋白甲基化的转录抑制。P21是DNA损伤后由抑癌基因*p53*诱导或p53过表达诱导的细胞周期依赖性激酶抑制剂，是HOTAIR的下游靶标。Cao等^[20]^研究发现通过siRNA诱导EZH2表观沉默，p21在NSCLC细胞中表达明显增加，这提示，通过与PRC2相互作用，HOTAIR可调节p21的表达。因此，HOTAIR调节LAD顺铂耐药的潜在机制可能与通过影响p21的表达而增加凋亡及G_0_期/G_1_期的细胞周期停滞有关。

AK126698，长度为3, 826 bp，存在于小脑中。研究^[21]^证明AK126698与NSCLC中的顺铂耐药有关。Yang等^[22]^发现AK126698在NSCLC的顺铂耐药中发挥重要作用。他们指出，AK126698与Wnt信号通路的多个成员有关，如NKD2和FZD8。β-catenin的过表达不仅可促进NSCLC的发生，还可促进化疗耐药^[23]^。同时，NKD2可通过与DVL结合抑制β-catenin^[24]^。A549细胞中AK126698的敲除可降低NKD2的表达，并增加β-catenin的表达。因此，AK126698可部分通过Wnt信号通路调节NSCLC中的顺铂耐药。AK126698的过表达可增加NSCLC对顺铂的敏感性。

HOTAIR及AK126698均参与NSCLC中的顺铂耐药，因此推测，他们可以作为潜在的治疗靶标。因为二者发挥相反的作用（HOTAIR的过表达促进顺铂耐药，而降低AK126698的表达可促进顺铂耐药），可利用相反的手段来抑制他们的作用。对于HOTAIR来说，采用有效的干扰来下调其表达可增加NSCLC患者对顺铂的敏感性，敏感化AK126698可成为缓解NSCLC患者顺铂耐药的有效治疗干预措施。然而，HOTAIR及AK126698调节化疗耐药的准确机制尚需进一步阐释。

### 2.2 肿瘤抑制性lncRNA

肿瘤抑制基因的失活在人类肿瘤的发病中发挥重要作用。因此，发现新的肿瘤抑制因子并阐释他们的功能是了解人类肿瘤启动潜在机制的重要过程，且对进一步研发新的治疗靶标非常重要。母系表达基因3（maternally expressed gene 3, MEG3）、生长停滞特异性蛋白6-反义RNA1（growth-arrest-specific gene 6-antisense RNA 1, GAS6-AS1）和BRAF激活的非编码RNA（BRAF activated non-coding RNA, BANCR）均为肿瘤抑制性lncRNA^[25]^，他们的下调可促进NSCLC的进展。研究这些lncRNAs为了解NSCLC的转移和进展提供了新的思路。

#### 2.2.1 MEG3

MEG3位于染色体14q32.3，长度约为1.6 kb，在各种正常组织中均表达。在多种肿瘤中可见MEG3表达的缺失，MEG3的再表达可抑制体外肿瘤的增殖。MEG3表达缺失的机制包括基因缺失、启动子甲基化等^[25-29]^。MEG3表达的缺失可促进不同肿瘤的进展，包括NSCLC。

Lu等^[30]^研究发现，与正常组织相比，NSCLC组织中MEG3的表达明显下降，尤其在晚期肿瘤及体积增加的肿瘤中。同时，MEG3的过表达可下调NSCLC的细胞增殖，诱导体外凋亡并阻止肿瘤发生。而且，MEG3正常或高表达患者的中位生存时间明显高于MEG3低表达水平的患者。MEG3的表达下降可导致p53蛋白的低水平表达。P53是一种转录因子，可调节各种基因的表达，这些基因可抑制肿瘤进展。一旦p53发生突变或呈低水平表达，即可促进肿瘤进展。综合来说，MEG3在NSCLC的发展中发挥重要作用。与Lu等^[30]^研究一致，另两个研究小组^[28, 29]^的研究发现MEG3可通过诱导p53的活化而发挥肿瘤抑制作用。Lu等^[30]^还发现，MEG3提示NSCLC患者的预后不良。

#### 2.2.2 GAS6-AS1

GAS6-AS1是一种肿瘤抑制lncRNA，位于染色体13q34。GAS6-AS1表达缺失的机制尚未被阐明；然而，GAS6-AS1的低表达或可促进不同肿瘤的进展，包括NSCLC。Han等^[31]^研究显示，与邻近正常组织相比，NSCLC组织中GAS6-AS1的表达明显下调，GAS6-AS1表达的下调与淋巴结转移呈负相关。GAS6-AS1表达的缺失与NSCLC的进展有关。潜在机制可能与影响其宿主基因*GAS6*有关。*GAS6*是Axl/Sky酪氨酸激酶家族的配体，是最先被确定的在生长停滞细胞中起诱导作用的基因。Axl的过表达可促进有丝分裂并在不同肿瘤中发挥促存活功能，还可介导神经胶质瘤细胞的增殖、迁移及侵袭^[32]^。GAS6对Axl有很高的亲和力，对于多种肿瘤的迁移和侵袭来说，GAS6/Axl是必需的。GAS6介导的细胞迁移和侵袭的机制可能为：通过AP-1活化蛋白1转录因子、c-Jun和ATF-2的JNK及ERK1/2依赖的机制，GAS6可诱导SLUG的表达，可降低E-cadherin的表达、诱导vimentin及细胞迁移^[33]^。GAS6-AS1位于GAS6的下游，在Han等^[31]^研究中，他们发现GAS6-AS1水平与GAS6 mRNA水平呈负相关。他们还发现在NSCLC患者中GAS6 mRNA水平与临床病理因素间存在相关性，GAS6水平的增加与淋巴结转移及TNM分期较晚呈正相关，提示预后不良，这显示，GAS6可调节GAS6-AS1的功能。

NSCLC与GAS6-AS1的下调有关，尤其在诊断为晚期的患者中，可成为NSCLC患者尤其是伴有转移患者的潜在诊断靶标。在Han等^[31]^研究中，单因素及多因素分析亦显示GAS6-AS1的表达是NSCLC患者总生存期的独立预后指标。

#### 2.2.3 BANCR

BANCR位于染色体9q21.11，大小为693 bp，可发挥肿瘤抑制作用，并可通过调节细胞生长停滞、抑制细胞侵袭而调节细胞增殖，从而降低恶性肿瘤的发病率。Flockhart等^[34]^在恶性黑色素瘤细胞中发现，BANCR在介导黑色素细胞瘤细胞迁移中发挥作用。

除黑色素瘤外，BANCR也可介导NSCLC的进展。Sun等^[35]^研究表明，与正常肺组织相比，NSCLC肿瘤组织中BANCR的表达水平显著下降，BANCR的异常表达与患者的总生存期相关：低BANCR表达水平患者的生存期显著短于高表达者。另外，Sun等^[35]^的研究显示上调BANCR的表达可抑制肿瘤细胞的侵袭和转移，而BANCR表达的敲除可促进细胞迁移和侵袭。

BANCR抑制NSCLC侵袭和转移的分子机制可能与上皮间质转化（epithelial-mesenchymal transition, EMT）有关。EMT的最主要特征为N-cadherin、vimentin的异常表达及E-cadherin的表达下调或缺失^[36-38]^。与EMT的主要特征相符合，BANCR的表达缺失可降低E-cadherin的表达，诱导N-cadherin、vimentin和基质金属蛋白酶（matrix metalloproteases, MMPs）。MMPs也参与细胞迁移，这提示EMT或许是BANCR介导NSCLC侵袭和转移的机制之一。BANCR的下调与NSCLC患者的肿瘤体积更大、病理分期更晚、转移距离更远和更短的生存时间有关^[35]^。因此，BANCR的异常表达为TNM分期提供了重要的预测价值，其或许可作为潜在的诊断和预后生物标志物。

## 3 结论

在所有的恶性肿瘤中，肺癌具有较高的发病率和死亡率。尽管许多研究人员致力于NSCLC发病机制的研究，然而潜在的机制仍未被完全阐明且治疗效果仍不理想。为进一步揭示NSCLC的机制并探索新的治疗方案，研究主要聚焦于lncRNA。LncRNA可作为新的诊断和预后生物标志物，并且可作为多种肿瘤的治疗靶标。对于NSCLC，lncRNA对于早期患者来说是极具希望的标志物，且可用作非侵袭性筛查。另外，lncRNA亦可作为化疗敏感性的预测标志物，利于提供更好的治疗选择。最后，lncRNA在NSCLC进展中的新兴作用为更深入的研究提供了良好的基础，且为研发更为有效的治疗药物提供了更好的途径。
